# Somatic *TEK* Mutation Identified in a Patient with Calvarial Venous Malformations

**DOI:** 10.3390/genes16101123

**Published:** 2025-09-23

**Authors:** Baojian Fan, Evan Dennis, Neel H. Mehta, William Davalan, Carla Fortes, Aditi Swamy, William Muñoz, Camilo Jaimes, Andrew T. Hale, Kristopher T. Kahle

**Affiliations:** 1 Department of Neurosurgery, Massachusetts General Hospital, Boston, MA 02114, USA; bfan@mgh.harvard.edu (B.F.); evan_dennis@hms.harvard.edu (E.D.); neelmehta@hms.harvard.edu (N.H.M.); william.davalan@mail.mcgill.ca (W.D.); cfortes@mgh.harvard.edu (C.F.); swamy.a@northeastern.edu (A.S.); wmunozmiranda@mgh.harvard.edu (W.M.); 2 Department of Radiology, Massachusetts General Hospital, Boston, MA 02114, USA; cjaimescobos@mgb.org; 3 Department of Neurosurgery, University of Alabama at Birmingham, Birmingham, AL 35294, USA; andrewthale@uabmc.edu; 4 Department of Human Biology and Neuroscience Institute, University of Cape Town, 7700 Cape Town, South Africa; 5 Broad Institute of MIT and Harvard, Cambridge, MA 02142, USA; 6 MGH Developmental Brain and CSF Disorders Program, Massachusetts General Hospital, Boston, MA 02114, USA; 7 Program in Neuroscience (PiN), Harvard University, Boston, MA 02138, USA

**Keywords:** calvarial vascular anomaly, pediatric neurosurgery, somatic mutation, *TEK*, venous malformation

## Abstract

**Background**: Calvarial venous malformations (VMs) are rare and genetically understudied. While somatic *TEK receptor tyrosine kinase* (*TEK*) mutations drive sporadic VMs, their role in scalp–calvarial VMs is unknown. We report the first pediatric case of a calvarial VM with a pathogenic somatic *TEK* mutation and its molecular implications. **Methods**: A 16-year-old female with a symptomatic parietal scalp VM underwent neurosurgical resection. Exome sequencing was performed on both lesional and blood DNA. Single-cell RNA sequencing (scRNA-seq) data from normal brain vasculature were analyzed for *TEK* expression and pathway enrichment. **Results**: A novel somatic *TEK* L914F mutation (chr9:27212760-C-T [GRCh38]), absent in germline DNA and population databases, was identified and predicted to be deleterious (CADD: 24). scRNA-seq data analysis revealed *TEK* enrichment in endothelial cells, particularly in fetal and arterial subtypes, and implicated angiogenesis and PI3K/Rho signaling as potential downstream phenotypic and molecular consequences. **Conclusions**: This first pediatric scalp VM with a somatic *TEK* L914F mutation expands the phenotypes associated with *TEK*-related vascular anomalies. These findings emphasize the role of somatic *TEK* mutation in diverse VMs and support genetic testing in sporadic cases. Further studies are needed to define therapeutic targets.

## 1. Introduction

Venous malformations (VMs) are congenital slow-flow vascular anomalies composed of venous vessels that lack the normal smooth muscle cell layer [1]. VMs are most often asymptomatic but may prompt investigation due to bluish discoloration of the skin or soft-tissue swelling. Rarely, VMs can enlarge during childhood or adolescence and may cause headaches, seizures, cognitive changes, or, in some cases, hemorrhage [2]. Scalp VMs can connect through transosseous emissary veins with intracranial dural sinuses; this phenomenon is known as sinus pericrania (SP), which is defined as an abnormal epicranial–dural venous connection. Accessory SP (draining only a portion of intracranial venous outflow) can be treated with surgical ligature or endovascular obliteration [3]. Intraosseous calvarial VMs are an uncommon subset of these lesions, characterized by expansion of the diploic space in cranial bones and often showing a distinctive “sunburst” trabecular pattern on imaging [4].

Genetic factors are believed to play a significant role in the development of VMs [5]. Previous studies have identified a key role for the TEK (TIE2) receptor in VM pathogenesis [6]. The *TEK receptor tyrosine kinase* (*TEK*) gene encodes the endothelial-specific receptor tyrosine kinase TIE2, which is crucial for angiopoietin-mediated signaling during vascular development [6]. TIE2 is primarily expressed in vascular endothelial cells and is crucial for the normal formation and maintenance of blood vessels. By interacting with angiopoietins, TIE2 supports endothelial cell survival and vessel maturation; disruptions in this pathway can alter vessel shape. Heterozygous germline *TEK* mutations lead to autosomal-dominant cutaneomucosal VM syndromes [7]. Notably, many sporadic VMs contain somatic activating *TEK* variants. For example, Limaye et al. found somatic *TEK* mutations in nearly half of sporadic VMs, with the most common being the L914F substitution [8]. These mutations cause ligand-independent TIE2 autophosphorylation, activating downstream signaling and leading to abnormal endothelial cell growth and dilation of venous channels. Although *TEK* is well known to be involved in cutaneous and mucosal VMs [7], *TEK* mutations have not been reported in calvarial VMs. Calvarial VMs linked to genetic changes are extremely rare.

Herein, we describe the first pediatric case of a calvarial VM with a somatic *TEK* L914F mutation. This case expands the known range of *TEK*-related vascular anomalies and highlights the importance of somatic genetic testing in VM cases.

## 2. Materials and Methods

### 2.1. Exome Sequencing and Data Analysis

Genomic DNA from the surgically resected VM lesional (somatic) tissue and blood or buccal swabs (germline) was isolated via previously described phenol–chloroform techniques [9] and was sequenced at the Yale Center for Genome Analysis using the standard protocol [10]. The sequencing depth of germline and somatic samples was at 150× and 500×, respectively. Somatic/germline single-nucleotide variants (SNVs) and small insertion–deletions (INDELs) were called using Sentieon 202308 (the TNseq algorithm for somatic variant calling and the DNAscope algorithm for germline variant calling) [11]. The VCF files generated by the pipeline were then normalized (left alignment of INDELs and splitting multiallelic sites into multiple sites) using bcftools 1.13 [12]. The raw variant calls were filtered following GATK best practices and consensus workflows [13]. Overlapped transcripts were identified for each variant, and the effects of the variants on the transcripts were predicted by Ensembl VEP (version 104) [14] and annotated with ANNOVAR (version 2025-3-2) [15]. Somatic calls were further filtered based on the variant allele frequency (VAF) in the tissue and blood, as well as the frequency of these variants in the ExAC and gnomAD databases (minor allele frequency [MAF] < 5%) [16].

### 2.2. Single-Cell RNA-Seq and Protein–Protein Interaction Analysis

The processing of the single-cell RNA-seq (scRNA-seq) atlas of the human brain vasculature has been described previously [17]. Briefly, endothelial cells were isolated using tissue digestion and FACS sorting of tissue samples. The vascular and perivascular cells were derived from the unsorted fraction. Cells from individual samples were integrated using the reciprocal PCA method and were subsequently clustered. Data were downloaded from the UCSC CellBrowser repository and loaded into Seurat (version 5.1) [18,19]. Differential expression between cell types, time points, and conditions was calculated with the Wilcoxon rank sum test using the FindAllMarkers command. Gene ontology (GO) analysis was conducted on the top 100 significantly differentially expressed genes between groups. These genes were expressed in at least 10% of cells in a given group and ranked by adjusted *p*-value (*Adj. P*). GO analysis was conducted with the GO Biological Process 2025 database using the EnrichR (version 3.4) package [20]. Protein–protein interactions of the top 100 differentially expressed genes among *TEK*-expressing endothelial cells were queried from the STRING database [21] using the stringApp (version 2.1.1) within Cytoscape (version 3.10.2). A confidence cutoff of 0.4 was applied. Hub genes were identified using the Maximum Clique Centrality (MCC) method within the cytoHubba app (version 0.1) [22]. Singletons and proteins without direct or indirect interactions with TEK were not visualized.

## 3. Results

### 3.1. Clinical Presentation

**Figure 1:**
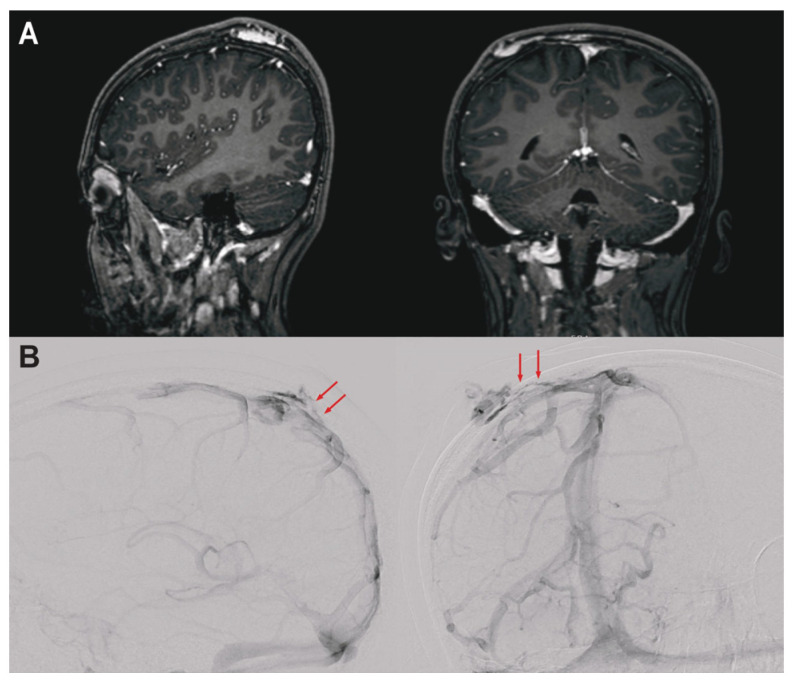
Pre-operative neuroimaging studies for a 16-year-old female with low-flow venous malformation. (**A**) Sagittal (left) and coronal (right) post-contrast T1-weighted MRI shows well-defined tortuous vessels in the right parietal scalp, suspicious for a venous malformation. The enhancement in the parietal bone overlying the superior sagittal sinus, in combination with subtle transdiploic tracks of linear enhancement, raised concern for patency of venous channels and a sinus pericranii. (**B**) Lateral and right anterior oblique digital subtraction angiography images following right internal carotid artery injection confirm a slow-flow venous lesion with modest blush and without evidence of arteriovenous shunting. The transcalvarial connections are apparent on both images (red arrows).

We present a case of a 16-year-old female with no prior medical conditions who was found to have a symptomatic right parietal scalp and calvarial VM measuring approximately 4.1 × 4.4 cm (AP × TV), with a few areas contiguous with calvarial emissary veins. The patient was born with a discoloration on the right parietal calvarium that was not associated with any other neurocutaneous findings. Following an unremarkable childhood and early adolescence, she developed severe, debilitating headaches (6–7/10), reported to occur monthly and worsen during menses. Initial brain magnetic resonance imaging (MRI, Figure 1A) and subsequent digital subtraction angiography confirmed serpiginous vessels overlying the right parietal calvarium without arteriovenous shunting, but with transcalvarial venous connections with the superior sagittal sinus (Figure 1B), consistent with a low-flow VM. She underwent an uncomplicated open resection of a right parietal scalp VM.

Notably, the patient’s mother is a 32-year-old woman with a known history of left frontal brain arteriovenous malformation (bAVM) who underwent preoperative embolization, left craniotomy, and eventually radiosurgical treatment for recurrent disease. Given the presentation of a low-flow extracranial VM in the context of a family history of recurrent bAVM, a familial AVM syndrome was suspected. The patient and her mother underwent germline whole-exome sequencing (WES), and the patient’s surgically resected VM tissue was sent for WES to enable somatic mutation analysis.

### 3.2. Integrative Genomic Analyses

WES analysis of the patient’s resected VM (somatic) tissue and matched germline blood identified 18 somatic variants, including 13 missense variants and 5 in-frame INDELs. Among these variants, five are located in genes (*EIF3E*, *NEMF*, *RALY*, *TEK*, and *TTC28*) that are highly expressed in the brain vasculature (Table 1). Only *TEK* has been linked to VMs (OMIM# 600195). The somatic mutation (chr9-27212760-C-T [GRCh38]; p.L914F) identified in *TEK* (pLI: 1; Mis-Z: 2.89) is not present in gnomAD and is predicted to be deleterious (CADD: 24; PolyPhen: 0.999; SIFT: 0). Moreover, the same mutation has been previously reported in sporadic VMs [8]. Therefore, the somatic mutation in *TEK* was the only pathogenic gene mutation in a mutation-intolerant gene (pLI > 0.9) that is also highly expressed in cerebral vasculature and has been previously implicated in a dominant OMIM disease with a relevant phenotype. No *TEK* gene variants were identified in the germline of either the patient or mother, ruling out a two-hit mechanism. Additionally, no compelling pathogenic transmitted gene variants were shared between them, suggesting that the same genetic factors do not cause the mother’s AVM and the patient’s calvarial VM.

**Figure 2:**
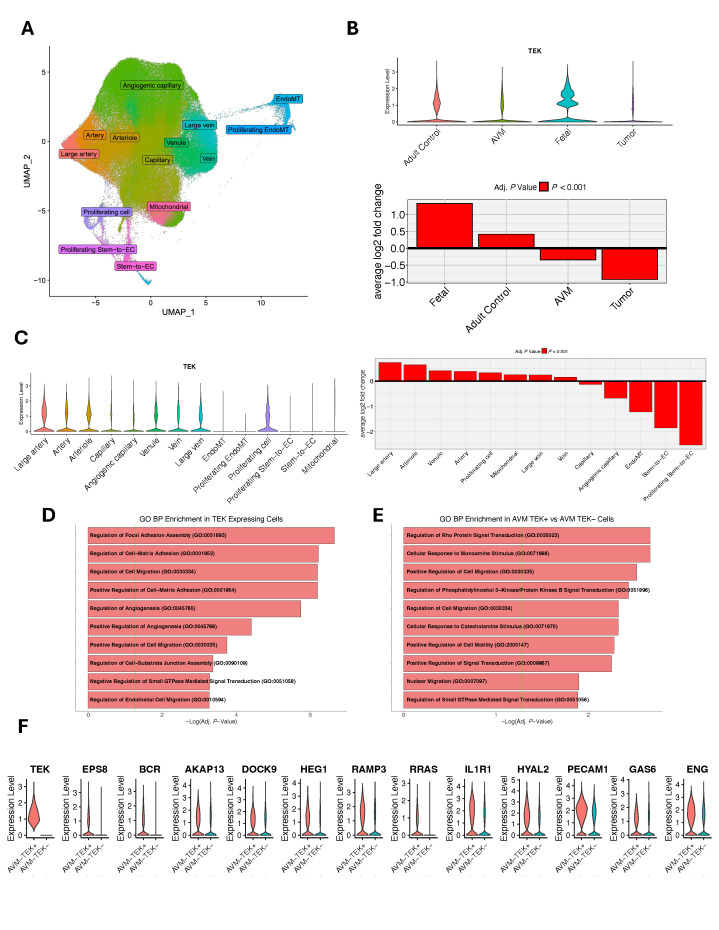
*TEK* expression in FACS-sorted fetal, adult control, AVM, and tumor endothelial cells. (**A**) UMAP (Uniform Manifold Approximation and Projection) plot of 243,521 FACS sorted endothelial cells colored by cell type rendered using Seurat (version 5.1) (see Methods). Endothelial-to-mesenchymal transition (EndoMT), endothelial cell (EC). (**B**) Violin plot and bar plot demonstrating *TEK* differential expression across conditions. (**C**) Violin plot and bar plot demonstrating *TEK* differential expression across endothelial cell types. Proliferating EndoMT cell ommited in bar plot to maintain axis scale (avg. log2 fold change = −4.22). (**D**) Top enriched Gene Ontology (GO) Biological Process terms among the top 100 differentially expressed genes (DEGs) in *TEK*-positive cells. The green vertical line indicates the threshold for significance. (**E**) Top enriched GO Biological Process terms among the top 100 DEGs in *TEK*-positive AVM cells vs. *TEK*-negative AVM cells. (**F**) Violin plots of top DEGs in *TEK*-positive vs. *TEK*-negative AVM cells included in GO terms related to Rho Protein and PI3K signaling pathways.

**Figure 3:**
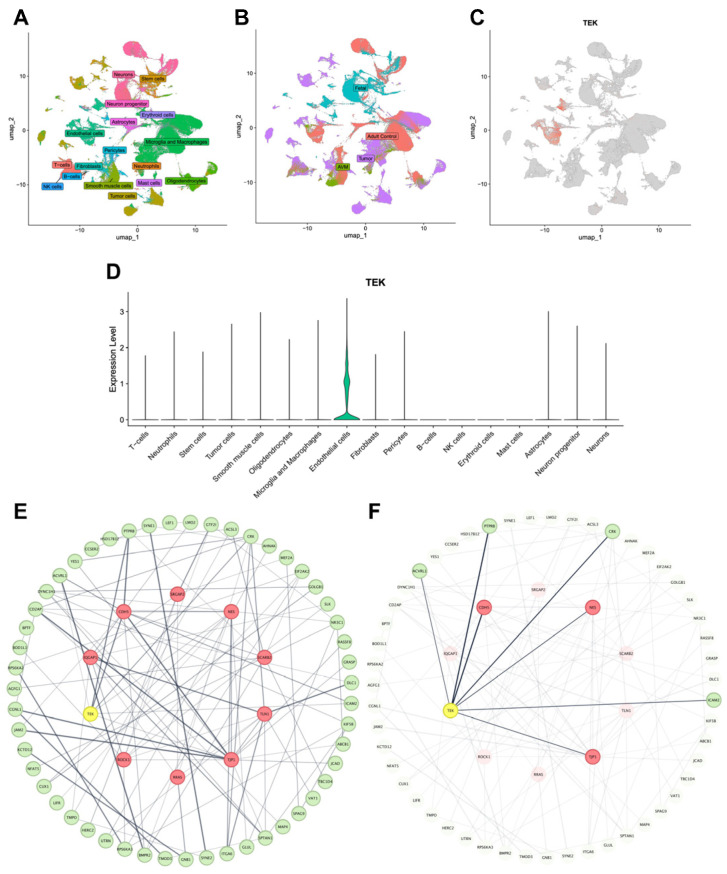
*TEK* expression in vascular and perivascular cells and the protein–protein interaction (PPI) network. (**A**,**B**) UMAP (Uniform Manifold Approximation and Projection) plot of fetal, adult control, AVM, and tumor vascular and perivascular cells colored by cell type (**A**), and condition (**B**). (**C**) UMAP plot of *TEK* expression among individual cells. (**D**) Violin plot of *TEK* expression across cell types. (**E**) PPI network of top differentially expressed genes among *TEK*-positive endothelial cells based on the STRING database. (**F**) Identical network highlighting first-degree neighbors of TEK. Nodes represent proteins and edges represent protein–protein interactions weighted by STRING confidence scores. The inner circle highlighted in yellow/red represents the top 10 hub proteins of increased connectivity in the network. TEK is a hub protein and is highlighted in yellow.

To gain a deeper understanding of how *TEK* may alter brain vasculature development and lead to pathology, we examined *TEK* expression in two scRNA-seq atlases comprising 212,214 unsorted vascular and perivascular cells and 243,521 FACS-sorted endothelial cells from fetal, adult, AVM, and tumor tissue samples [17] (Figure 2A and Figure 3A,B). In all vascular and non-vascular cell types in the unsorted atlas, *TEK* showed particularly specific expression in endothelial cells (Figure 3C,D). In the FACS-sorted endothelial atlas, *TEK* was most highly expressed in fetal endothelial cells (Figure 2B). Additionally, *TEK* was significantly upregulated in endothelial cells of large arteries, arterioles, and venules, with lower expression in capillary cell types (Figure 2C). GO analysis of the top differentially expressed genes among *TEK*-positive cells revealed enrichment in pathways related to the regulation of focal adhesion assembly (GO: 0051893, *Adj. P* = 2.23 × 10^−7^), cell migration (GO: 0030334, *Adj. P* = 6.32 × 10^−7^), and angiogenesis (GO: 0045765, *Adj. P* = 1.78 × 10^−6^) (Figure 2D).

Protein–protein interaction analysis of these genes uncovered a strong connectivity network (Figure 3E). TEK ranked among the top 10 hub proteins within this network and directly interacted with three other hub proteins vital for endothelial development and function: NES, CDH5, and TJP1 (Figure 3F). Lastly, to explore the role of *TEK* in AVMs, we analyzed differentially expressed genes between *TEK*-positive and *TEK*-negative AVM endothelial cells. GO analysis of the top 100 genes differentially expressed between these groups showed enrichment in pathways involving regulation of Rho protein signal transduction (GO: 0035023, *Adj. P* = 2.15 × 10^−3^) and PI3K signal transduction (GO: 0051896, *Adj. P* = 3.67 × 10^−3^), driven by *TEK* along with *EPS8*, *BCR*, *AKAP13*, *DOCK9*, and *HEG1*, and *RAMP3*, *RRAS*, *IL1R1*, *HYAL2*, *PECAM1*, *GAS6*, and *ENG*, respectively (Figure 2E,F). These findings suggest that dysfunction in these pathways, which are crucial for cell growth and migration, may contribute to *TEK*-related VM development.

## 4. Discussion

Herein, we present a case of a 16-year-old female with a scalp VM associated with a predicted damaging somatic *TEK* L914F mutation (Table 1). While this mutation has been identified in sporadic VMs [8], to our knowledge, this is the first description of a scalp VM harboring this mutation. These data highlight the variable phenotypes that may arise from somatic *TEK* mutations. Germline *TEK* mutations have been reported to cause a rare, inherited form of venous anomaly known as mucocutaneous venous malformation [7]. Eight somatic *TEK* mutations have been identified in lesions from 28 (49.1%) of 57 individuals with sporadic VMs, including a frequent L914F mutation from 24 (42.1%) of 57 individuals [8]. However, neither germline nor somatic *TEK* mutations have been reported in human patients with scalp VMs.

In this study, the proband’s calvarial VM manifested as a congenital parietal scalp discoloration, evolving into debilitating headaches exacerbated during menses—a feature potentially linked to hormonal modulation of vascular tone or endothelial proliferation. Unlike typical cutaneous VMs, calvarial lesions may involve transosseous emissary veins, creating a risk of sinus pericranii and intracranial communication, as evidenced by venous drainage into the superior sagittal sinus (Figure 1) [3]. This anatomical specificity highlights the importance of meticulous neuroimaging (e.g., MRI/angiography) to distinguish calvarial VMs from other vascular anomalies and inform surgical planning. Familial clustering with the mother’s bAVM raises questions about shared genetic susceptibility, though germline analysis ruled out pathogenic variants in *TEK* or other established vascular genes, favoring a somatic-driven mechanism in this case. Indeed, integration of somatic genetic testing with MRI and MRA may help provide further diagnostic clarity to the broad diagnostic differential of intracranial and calvarial vascular formations, ranging from high-flow arteriovenous malformations to slow-flow lymphatic malformations [23,24].

scRNA-seq revealed preferential expression of *TEK* in fetal endothelial cells and arterial/venular subtypes (Figure 2), aligning with its role in vascular maturation [8,25]. Protein–protein interaction networks positioned TEK as a hub protein directly interacting with NES, CDH5, and TJP1, all critical for endothelial integrity and angiogenesis [26,27,28]. Pathway enrichment highlighted dysregulation of PI3K and Rho GTPase signaling, which regulate endothelial cell migration, cytoskeletal reorganization, and vascular permeability [6,7]. Dysfunctional PI3K signaling may drive abnormal endothelial proliferation, while alterations in the Rho pathway could impair vessel stabilization, collectively contributing to VM pathogenesis. Future directions could include utilizing tools to detect somatic mutations at a single-cell resolution to compare endothelial cells harboring *TEK* L914F to *TEK* wild-type endothelial cells.

*TEK* mutations (including L914F) have been shown to induce venous malformations in more than 75% of zebrafish embryos, which closely recapitulate the clinical features observed in human patients [29]. Overexpression of the L914F mutant in human umbilical vein endothelial cells showed abnormal localization and ligand response, unlike wild-type *TEK* and the R849W mutant [8]. This indicates that different *TEK* mutations may have distinct effects. Preclinical studies in zebrafish demonstrate that mTOR inhibitors (e.g., sirolimus/rapamycin) suppress *TEK*-driven VM formation by targeting PI3K/AKT downstream signaling [29]. While sirolimus is clinically used for complex vascular anomalies [7], its efficacy in calvarial VMs remains unexplored. This case underscores the potential utility of mTOR inhibition in *TEK*-mutant VMs, particularly for lesions refractory to surgery. Furthermore, the fact that germline *TEK* variants with vascular relevance are less common than somatic *TEK* variants underscores the importance of somatic profiling in guiding targeted therapies.

Since *TEK*-related VM can be presented in two ways—autosomal dominant germline or somatic (mosaic) variants—genetic counseling can differ between carriers of germline and somatic mutations [7]. Most probands with a germline mutation have an affected parent; however, the rate of de novo variants is unknown. Each child of an affected person has a 50% chance of inheriting a germline mutation. Prenatal testing is possible if the *TEK* variant is identified. In contrast, VMs caused by somatic mutations are not inherited. Sibling recurrence has not been reported, and the risk to siblings is comparable to that of the general population. Due to mosaicism, the risk of offspring transmission is less than 50%.

This study has several limitations. Primarily, it describes a single pediatric case, limiting the generalizability of the findings and precluding assessment of *TEK* mutation prevalence in calvarial VMs. The pathogenic impact of the somatic *TEK* L914F mutation was inferred computationally and from prior literature but lacked direct functional validation in patient-derived cells. Mechanistic insights into PI3K/Rho signaling relied on pre-existing, non-patient-specific scRNA-seq datasets, which may not fully reflect the calvarial VM microenvironment. Nonetheless, the inclusion of single-cell data is merely supportive, demonstrating that *TEK* is expressed in the relevant human cell type that would be affected by the somatic mutation. Finally, while mTOR inhibition was suggested as a potential therapy based on preclinical data, no clinical efficacy data for this approach in calvarial VMs were generated. Larger cohorts and functional studies are needed to validate these findings.

## 5. Conclusions

This study reports the first pediatric case of a calvarial VM harboring a somatic *TEK* L914F mutation, thereby expanding the phenotypic spectrum of *TEK*-related vascular anomalies. The somatic variant, absent in germline DNA and population databases, highlights the role of somatic *TEK* mutations in various VM subtypes, including anatomically distinct calvarial lesions. Single-cell transcriptomics showed *TEK* enrichment in fetal and arterial/venular endothelial cells, with pathway analyses indicating dysregulated angiogenesis, focal adhesion, and PI3K/Rho signaling in VM development. Germline analysis of the proband and her mother (who has a history of bAVM) did not identify pathogenic variants in *TEK* or other established vascular genes, suggesting a somatic-driven mechanism in this case. These findings support genetic testing in sporadic vascular anomalies to improve diagnosis and counseling. Further research is needed to understand the role of *TEK* in calvarial VMs and assess targeted therapies, such as mTOR inhibitors, for this understudied disorder.

## Figures and Tables

**Table 1:** Candidate somatic variants.

Gene	Variant (GRCh38) ^1^	Amino Acid Change	VAF	MAF (gnomAD)	CADD	Mis-Z	pLI
*EIF3E*	8-108217434-C-T	p.C250Y	0.0138	0	31.0	3.10	0
*NEMF*	14-49785100-T-C	p.K1022R	0.0231	6.2 × 10^−7^	23.8	1.29	0
*RALY*	20-34077060-G-A	p.G231S	0.0115	0.0065	0.035	1.45	0.03
***TEK *** **^2^**	**9-27212760-C-T**	**p.L914F**	**0.0339**	**0**	**24.0**	**2.89**	**1**
*TTC28*	22-28679648-G-T	p.P26T	0.0600	0.033	14.3	3.47	1

^1^ All variants are heterozygous and missense single-nucleotide variants (SNVs). CADD: Combined Annotation Dependent Depletion; gnomAD: The Genome Aggregation Database; MAF: Minor Allele Frequency; Mis-Z: Z-scores of the observed missense counts compared to expected; pLI: Probability of Loss of Function Intolerance. VAF: Variant Allele Frequency. ^2^ The somatic mutation in *TEK* was the only pathogenic gene mutation in a mutation-intolerant gene (pLI > 0.9) that is also highly expressed in cerebral vasculature and has been previously implicated in a dominant OMIM disease with a relevant phenotype.

## Data Availability

Single-cell RNA-seq data were downloaded from the UCSC CellBrowser repository: https://cells.ucsc.edu/?ds=brain-vasc (accessed on 5 May 2025); Gene Ontology data were downloaded from the GO Biological Process 2025 database: https://www.geneontology.org/docs/ontology-documentation/ (accessed on 8 May 2025); and protein–protein interactions data were queried from the STRING database: https://string-db.org/cgi/input?sessionId=biLdHcEMESIm&input_page_show_search=on (accessed on 6 May 2025).

## Abbreviations

The following abbreviations are used in this manuscript:

*Adj. P*	Adjusted *P*-value
bAVM	Brain arteriovenous malformation
CADD	Combined annotation-dependent depletion
gnomAD	The genome aggregation database
INDELs	Insertion–deletions
MAF	Minor allele frequency
MCC	Maximum clique centrality
Mis-Z	Z-scores of the observed missense counts compared to expected
MRI	Magnetic resonance imaging
pLI	Probability of loss of function intolerance
scRNA-seq	Single-cell RNA sequencing
SNVs	Single-nucleotide variants
SP	Sinus pericrania
*TEK*	*TEK receptor tyrosine kinase*
VAF	Variant allele frequency
VM	Venous malformation
WES	Whole-exome sequencing
